# Quality of life and the prevalence of urinary incontinence after surgical treatment for gynecologic cancer: a questionnaire survey

**DOI:** 10.1186/s12905-020-01012-7

**Published:** 2020-07-17

**Authors:** Noriko Nakayama, Tetsuya Tsuji, Makoto Aoyama, Takafumi Fujino, Meigen Liu

**Affiliations:** 1 Department of Rehabilitation, Teine Keijinkai Hospital, Sapporo, Japan; 2 Department of Rehabilitation Medicine, Keio University School of Medicine, Tokyo, Japan; 3 Department of Obstetrics & Gynecology, Teine Keijinkai Hospital, Sapporo, Japan

**Keywords:** Gynecologic cancer, Complications, Urinary incontinence, Quality of life

## Abstract

**Background:**

Although there have been a number of reports on urinary voiding symptoms associated with surgical interventions for gynecologic cancer and post-voiding symptoms, there have been few reports on urinary storage symptoms such as urinary incontinence (UI) and overactive bladder (OAB). The purpose of this study was to examine the rates and impact on quality of life (QOL) of urinary storage symptoms after gynecologic cancer surgery.

**Methods:**

A questionnaire survey, including Japanese-language versions of the International Consultation on Incontinence Questionnaire-Short Form (ICIQ-SF), Overactive Bladder Symptom Score (OABSS), and Incontinence Impact Questionnaire-7 (IIQ-7), was distributed to gynecologic cancer patients who underwent hysterectomy between 2008 and 2013.

**Results:**

Of the 145 patients analyzed, 49 (33.8%) had UI pre-surgery, and 76 (52.4%) had UI post-surgery, including 34 (35.4%) first-time UI patients, with a significant difference between pre- and post-surgery. Of the 49 subjects with UI pre-surgery, 43 (87.7%) had stress incontinence, while of the 76 patients with UI post-surgery, 44 (57.1%) had stress incontinence, and 24 (31.2%) had mixed incontinence. Seven (4.8%) subjects had OAB pre-surgery, whereas 19 (13.1%) had OAB symptoms post-surgery (including 15 first-time OAB patients), with a significant difference between pre- and post-surgery. IIQ-7 scores were markedly higher for patients with mixed incontinence post-surgery than for those with stress incontinence, indicating a lower QOL. Logistic regression analysis identified the number of Cesarean sections and days of urinary bladder catheterization as risk factors for postoperative UI.

**Conclusions:**

UI and OAB rates were higher after gynecologic cancer surgery than in the general female population. The mixed incontinence rate was markedly higher post-surgery; QOL was low for such patients due to the combination of urge and stress incontinence. Multiple Cesarean sections and urinary bladder catheterization post-surgery were risk factors for post-surgical UI.

## Background

As of 2012, the number of patients in Japan diagnosed with gynecologic cancer was 10,908 for cervical cancer, 13,606 for endometrial cancer, and 9384 for ovarian cancer [1] (whereas the number for peritoneal cancer is unknown because it is a relatively rare cancer [2]), and these numbers are increasing each year. Treatment outcomes have improved along with advances in medical technology: the 5-year relative survival rate for cases diagnosed between 2006 and 2008 was 73.4% for cervical, 81.1% for endometrial, and 58.0% for ovarian cancers [3], with the number of patients surviving during and after treatment for gynecologic cancer increasing. Therefore, the decrease in the quality of life (QOL) of patients due to complications associated either with the disease itself or its treatment is gaining in importance.

One common complication after surgical intervention for gynecologic cancer is lower urinary tract symptoms. The reported frequency varies from 12.2 to 51% [4–6], which is high enough to suggest a marked impact on QOL. Lower urinary tract symptoms can be classified into urinary storage, urinary voiding, and post-voiding symptoms [7]. Urinary storage represents an obstacle to maintaining urine and is further divided into urinary incontinence (UI) and overactive bladder (OAB). Voiding symptoms are symptoms related to urinary excretion, such as the loss of urinary urgency, urinary retention, urinary decrease, and so on. Post-voiding symptoms include residual urine symptoms after urination.

Although there have been a number of reports on urinary voiding symptoms associated with surgical interventions for gynecologic cancer [8] and post-voiding symptoms [9], there have been few reports on urinary storage symptoms. Furthermore, the previous studies did not analyze the differences in the prevalence of UI and OAB pre- and post-surgery. The purpose of this study was to investigate, by questionnaire survey, urinary storage symptoms in gynecologic cancer patients to clarify the prevalence of such symptoms (i.e., UI and OAB) pre- and post-surgical intervention and examine their impact on patients’ QOL.

## Methods

### Study design

This was a cross-sectional study. The sample size was calculated using a test for independence with sample-size calculating software (G*Power version 3.1.9.4 for Windows; http://www.gpower.hhu.de). A questionnaire survey was distributed by mail to women meeting the following criteria: a diagnosis of cervical, endometrial, ovarian, or peritoneal cancer and subsequent treatment by hysterectomy at Teine Keijinkai Hospital between 2008 and 2013. Exclusion criteria were: 1) under 20 years of age; 2) a history of tumor involving the urinary organs; 3) a fistula of the vagina, rectum, or bladder; or 4) an inability to understand the questionnaire survey. The evaluation method for urinary storage symptoms (i.e., UI or OAB) used the Japanese versions of the International Consultation on Incontinence Questionnaire–Short Form (ICIQ-SF) [10], the Overactive Bladder Symptom Score (OABSS) [11], and the Incontinence Impact Questionnaire-7 (IIQ-7) [12].

### Outcome assessment

Medical records were reviewed to extract data for the following items: age, degree of obesity (body mass index, BMI), number of pregnancies, vaginal deliveries, Cesarean sections, miscarriages (including induced abortions), inpatient days, period from operation to questionnaire response, diagnosis, operation type [13] (adnexal preservation/non-preservation, pelvis, hypogastric nerve preservation/non-preservation, lymphadenectomy, operative time, and blood loss at the time of surgery), and days of urinary bladder catheterization post-surgery.

#### International consultation on incontinence-questionnaire–short form (ICIQ-SF) [10]

The ICIQ-SF is an evaluation tool for incontinence classification and determination of severity and type (stress, urge, mixed, and overflow incontinence) [14–16]. The Japanese version of the ICIQ-SF [17] was used to evaluate symptoms pre- and post- surgery.

#### Overactive bladder symptom score (OABSS) [11]

This questionnaire is a tool developed for the diagnosis of OAB, as well as for the determination of its severity and type [18]. The Japanese version of the OABSS was used to evaluate symptoms pre- and post-surgery.

#### Incontinence impact Questionnaire-7 (IIQ-7) [12]

This tool is used to evaluate patient QOL based on the psychosocial effects of urologic problems in everyday life [19, 20]. The Japanese version of the IIQ-7 was used to evaluate symptoms post-surgery.

### Statistical analyses

The Kolmogorov-Smirnov test was used to test the distributions of the results of the pre- and post-surgery UI groups for normality, and it showed that they were not normally distributed. Therefore, nonparametric tests were used to compare the results of the pre- and post-surgery UI groups. The unpaired *t*-test was used to compare the medical information of subjects analyzed and the uncollected questionnaires from non-responders to determine whether there was any potential bias that might affect the results. The test of independence for each operation type was used to analyze the presence/absence of UI pre- and post-surgery, and this was compared among the four groups. In addition, the chi-squared test was used to compare the presence/absence of UI pre- and/or post-chemotherapy and/or radiotherapy for each diagnosis.

McNemar’s test was used to analyze differences in the prevalence of both UI and OAB pre- and post-surgery, and Wilcoxon’s rank-sum test was used to compare the frequency and volume of UI and the effect on QOL based on the responses to the ICIQ-SF. In addition, the number of people classified into each of the 4 groups based on the degree of UI pre- and post-surgery was evaluated. Total OABSS scores were compared pre- and post-surgery using Wilcoxon’s rank-sum test. The total and subdomain scores for the IIQ-7 questionnaire were compared using the Kruskal-Wallis rank test based on the 4 classifications of the ICIQ-SF (stress, urge, mixed, and overflow).

Furthermore, stepwise logistic regression analysis was performed using basic and medical data as independent variables, and the presence/absence of UI pre- and post-surgery as a dependent variable to identify the factors related to UI post-surgery.

SPSS statistics version 21 (IBM SPSS, Chicago, IL) was used for all statistical analyses, with the significance level set at 5%.

## Results

**Fig. 1:**
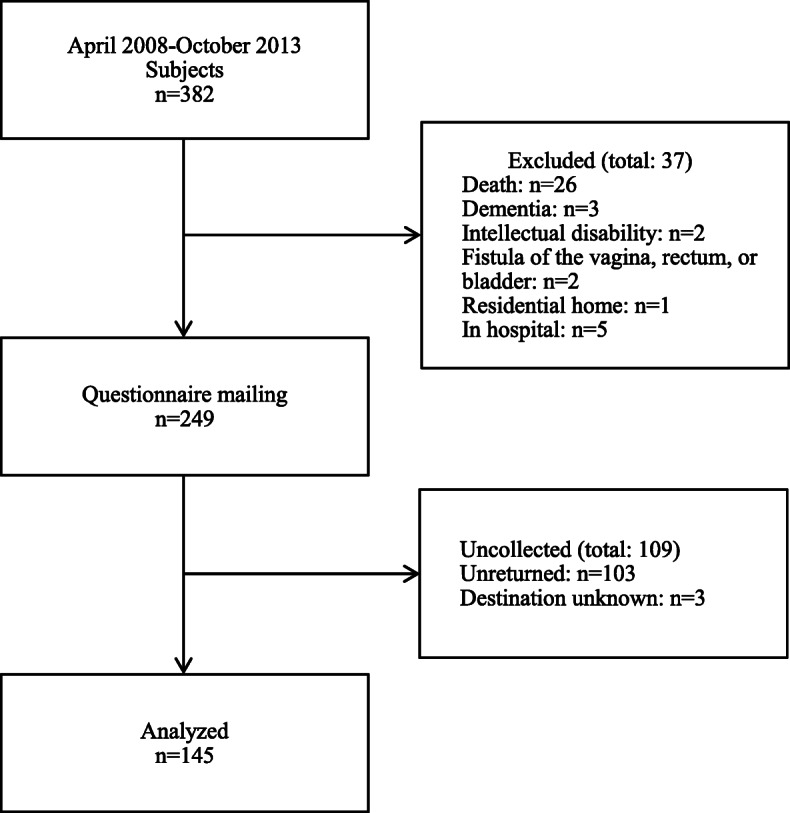
Flowchart of the inclusion process for subjects

A total of 382 women were treated at Teine Keijinkai Hospital for cervical, endometrial, ovarian, or peritoneal cancer between April 2008 and October 2013. However, 133 were excluded based on the above exclusion criteria, so the questionnaire survey was sent to 249 women. Of these, responses (together with consent) were received from 145 women (response rate 58.2%), and these 145 women were included in the analysis (Fig. 1). Age and number of vaginal deliveries were significantly higher in the subjects analyzed than in the non-responders. The patients’ background characteristics and medical data are shown in Table 1. Patient medical background by diagnosis is shown in Table 2.

**Table 1:** Patients’ background characteristics and medical data

Age at questionnaire survey (y), mean ± SD (range)	59.0 ± 12.0 (31–89)
Inpatient days	17.2 ± 11.1 (3–56)
BMI (kg/m^2^)	23.2 ± 3.8 (14.9–35.0)
Period from operation to questionnaire response (days)	839.6 ± 48.6 (10–1918)
Pregnancies, mean ± SD (range)	2.2 ± 1.5 (0–6)
Vaginal deliveries	1.6 ± 1.2 (0–5)
Cesarean sections	0.2 ± 0.5 (0–3)
Miscarriages	0.4 ± 0.7 (0–3)
Diagnosis, %, (n)	
Cervical cancer	30.3 (44)
Endometrial	40.0 (58)
Ovarian	25.5 (37)
Peritoneal	2.8 (4)
Rectal (infiltration cancer of uterus)	1.4 (2)
Operation type, %, (n)	
Radical hysterectomy	15.9 (23)
Modified hysterectomy	39.3 (57)
Simple hysterectomy	15.2 (22)
Laparoscopic hysterectomy	29.7 (43)
Adnexa non-preservation	81.4 (118)
Pelvis, hypogastric nerve non-preservation	9.0 (13)
Lymphadenectomy	60.0 (87)
Operative time (minutes), mean ± SD (range)	867.0 ± 988.8 (43–654)
Blood loss at time of surgery (ml), mean ± SD (range)	269.6 ± 138.6 (5–4254)
Days of urinary bladder catheterization post-surgery, mean ± SD (range)	4.9 ± 2.7 (1–15)

**Table 2:** Patients’ medical background characteristics by diagnosis

Cervical cancer (*n* = 44)	
Histological diagnosis, %, (n)	
Adenocarcinoma	20.5 (9)
Squamous cell carcinoma	77.3 (34)
Unknown	2.3 (1)
FIGO classification (2008), %, (n)	
Stage I	77.3 (34)
Stage II	15.9 (7)
Stage III	4.5 (2)
Stage IV	2.3 (1)
Endometrial (*n* = 58)	
Histological diagnosis, %, (n)	
Adenocarcinoma	89.7 (52)
Squamous cell carcinoma	1.7 (1)
Unknown	6.9 (4)
FIGO classification (2008), %, (n)	
Stage I	75.9 (44)
Stage II	3.4 (2)
Stage III	15.5 (9)
Stage IV	0 (0)
Unknown	5.2 (3)
Ovarian (*n* = 37)	
Histological diagnosis, %, (n)	
Adenocarcinoma	78.4 (29)
Squamous cell carcinoma	5.4 (2)
Unknown	16.2 (6)
FIGO classification (2008), %, (n)	
Stage I	54.1 (20)
Stage II	5.4 (2)
Stage III	13.5 (5)
Stage IV	16.2 (6)
Unknown	10.8 (4)
Peritoneal (*n =* 4)	
Histological diagnosis, %, (n)	
Adenocarcinoma	75.0 (3)
Squamous cell carcinoma	0 (0)
Unknown	25.0 (1)
FIGO classification (2008), %, (n)	
Stage III	50.0 (2)
Stage IV	50.0 (2)

Rectal (infiltration by cancer of the uterus) (*n* = 2)

### ICIQ-SF

Patients who selected a response other than “0” on Question 1 related to the frequency of UI were regarded as positive for UI. Of the 145 subjects from whom responses were received, pre-surgical UI was present in 49 (i.e., score ≥ 3 for Q3 + 4 + 5; prevalence 33.8%). On the other hand, post-surgical UI was present in 52.4% (76/145), including 35.4% (34/96) with UI for the first time. There was a significant difference in the prevalence of UI between pre- and post-surgery. There was no significant difference in age between those with and without UI either pre- or post-surgery. There was no significant difference in the incidence of UI post-surgery by cancer site (cervical, endometrial, ovarian, or peritoneal) between operation types or the presence or absence of pre- and/or post-chemotherapy and/or radiation therapy.

The frequency and volume scores of UI were 1.4 ± 0.8 and 2.1 ± 0.7, respectively, pre-surgery, while the impact of incontinence on daily life was 1.3 ± 1.4. The scores post-surgery were 1.9 ± 1.5, 2.7 ± 1.7, and 2.3 ± 2.5, respectively, with significant differences between pre- and post-surgery.

**Fig. 2:**
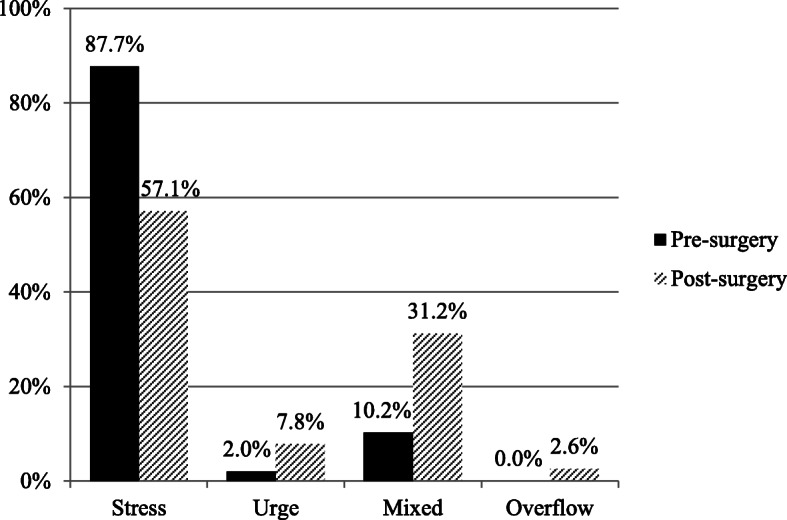
Classification of UI pre- and post-surgery. The vertical axis shows the rate of incontinence, and the horizontal axis shows the UI classification. The rate of stress incontinence is significantly higher than that of either urge or overflow incontinence. Compared to pre-surgery, the post-surgery rate of stress incontinence is significantly lower, while the rate of mixed incontinence is significantly higher

Regarding UI classification, of the 49 subjects who had incontinence pre-surgery, 43 had stress incontinence (87.7%), 1 had urge incontinence (2.0%), 5 had mixed incontinence (10.2%), and 0 (0%) had overflow incontinence (Fig. 2). The rate of stress incontinence was significantly greater than that of either urge or mixed incontinence. On the other hand, of the 76 subjects with UI post-surgery, 44 had stress incontinence (57.1%), 6 had urge incontinence (7.8%), 24 had mixed incontinence (31.2%), and 2 (2.6%) had overflow incontinence. The rate of stress incontinence was significantly higher than that of either urge or overflow incontinence. Compared to pre-surgery, the rate of stress incontinence was significantly lower, while the rate of mixed incontinence was significantly higher.

### OABSS

Seven subjects (4.8%) had OAB based on the OABSS questionnaire responses pre-surgery. However, 19 subjects (13.1%) had OAB post-surgery, including 15 (10.3%) with OAB for the first time. There was a significant difference in the prevalence of OAB between pre- and post-surgery.

### IIQ-7

The median (range) total score for subjects with UI post-surgery was 4.0 (0.0–18.0), and the subscale scores were 4.2 (0.0–18.0) for physical activity, 7.8 (0.0–18.0) for travel, 9.8 (1.0–18.0) for social/relationships, and 3.5 (2.0–5.0) for emotional health (each total score out of 21).

The scores for urinary incontinence for the 4 classifications of the post-surgery IIQ-7 are shown in Table 3. There was a significant difference among the 4 classifications. For all total and subdomain scores, the mixed score was significantly higher than the stress incontinence score (each subscale score out of 100) (Table 3).

**Table 3:** IIQ-7 scores for the four types of urinary incontinence post-surgery

IIQ-7 Subscale scores	Stress (*n =* 44)	Urge (*n* = 6)	Mixed (*n* = 24)	Overflow (*n =* 2)	χ^2^	*P*-value	Multiple comparisons
Physical activity	16.7 (0.0–83.3)	33.3 (0.0–49.9)	33.3 (0.0–83.3)	0.0 (0.0–0.0)	9.7	0.021	Stress< Mixed
Travel	8.33 (0.0–99.9)	33.3 (0.0–99.9)	50.0 (0.0–99.9)	25.0 (0.0–49.9)	11.9	0.008	Stress< Mixed
Social/ Relationships	0.0 (0.0–66.6)	33.3 (0.0–99.9)	66.6 (0.0–99.9)	66.6 (66.6–66.6)	19.5	0.000	Stress< Mixed
Emotional health	16.7 (0.0–66.6)	33.3 (0.0–99.9)	33.3 (0.0–99.9)	0 (0.0–0.0)	16.1	0.001	Stress< Mixed
Total score	4.2 (0.0–18.0)	7.8 (0.0–18.0)	9.6 (1.0–18.0)	3.5 (2.0–5.0)	16.8	0.001	Stress< Mixed

Values are medians (range) unless otherwise indicated

### Factors related to postoperative UI

The logistic regression analysis for the presence/absence of onset of UI post-surgery showed that the number of Cesarean sections (OR 2.4, CI 1.1–5.5) and days of urinary bladder catheterization (OR 1.2, CI 1.1–1.4) were risk factors for postoperative UI.

## Discussion

### Prevalence of pre- and post-surgical UI – a comparison by incontinence classification

Many previous studies have used the Urogenital Distress Inventory (UDI) as a tool for the evaluation of UI classifications. Although the UDI can be used to classify UI, it cannot be used to evaluate the frequency or volume of UI. On the other hand, the ICIQ-SF is a new questionnaire developed by the International Consultation on Incontinence (ICI). Since it can also be used to evaluate the frequency and volume of UI, it can be used for all UI patients regardless of sex or age. The final version, after verification of its reliability and validity, was released in 2001, with a Japanese version developed by Goto et al. [17]. The prevalence of UI among women in Japan has been reported to range between 26 and 53.7% [21–23].

In the present study, evaluation using the ICIQ-SF showed that the prevalence of UI pre-surgery was 33.8%, which is comparable to the previously reported figures. On the other hand, the prevalence of UI post-surgery was 52.4%, with a significant increase noted after surgical intervention. Hazewinkel et al. reported the prevalence of UI post-surgery to be 24% [24] based on a questionnaire survey distributed by mail to 146 cervical cancer patients after radical hysterectomy, a value much lower than that observed in the present study. The reasons for this discrepancy are thought to be that the subjects in their study were younger than those in the present study, the median time from surgery to questionnaire was 6 (range, 1 to 11) years, which was longer than that in the present study, and the method of UI evaluation also differed.

As mentioned above, the prevalence of UI post-surgery in the present study was 52.4%, of which 34.4% of the patients newly experienced UI post-surgery. The study by Hazewinkel et al. [24] did not compare subjects with UI pre- and post-surgery, and the present study is the first to identify patients newly experiencing UI post-surgery.

With regard to incontinence classification, the ICIQ-SF was used for classification in the present study. Results showed that 87.7% of those with UI had stress incontinence, 10.2% had mixed incontinence, 2.0% had urge incontinence, and 0.0% had overflow incontinence pre-surgery, with the majority of patients having stress incontinence. Araki et al. conducted a questionnaire survey of working women [25] and reported that the prevalence of women with UI was 16.7%, categorized as 72.7% with stress incontinence, 12.1% with urge incontinence, and 9.9% with mixed incontinence. Their results were quite similar to the present results. On the other hand, the rates by classification were 57.1, 7.8, 31.3, and 2.6% for stress, urge, mixed, and overflow incontinence, respectively. The rate for stress incontinence was markedly lower, whereas that for mixed incontinence was markedly higher than the pre-surgery values.

Stress incontinence accounts for the majority of cases of UI among women in general, and it has been reported to be caused by aging (over 40 years of age) and the tendency for pelvic floor muscles to become weaker due to obesity [26]. However, there was no significant difference in age between those with and without UI either pre-or post-surgery in the present study, and this is likely because the UI in the present study population was due to surgical invasion rather than age. On the other hand, in gynecological cancer patients, tissues supporting the cervix, such as the vesico-uterine ligament and the cervico-uterine ligament, are separated from the uterine cervix with removal of the uterus [13], leading to collapse of the pelvic mechanism balance and the bladder or urethra, relaxation of the pubococcygeus muscle, and insufficient closure of the urethra, resulting in the onset or exacerbation of stress incontinence [27]. In addition, it is thought that, in cases where the hypogastric nerve (a sympathetic nerve) is damaged during hysterectomy [13], the pelvic nerve (a parasympathetic nerve) becomes dominant, resulting in the occurrence of urinary urgency [28], i.e., OAB symptoms, subsequently leading to mixed (including stress) incontinence.

### Prevalence of pre- and post-surgical OAB

The OAB-q [29] and OABSS [18] are two evaluation tools for OAB for which the reliability and validity have been verified. The OAB-q consists of 8 items regarding symptoms and 25 items for QOL, but it has a major drawback in that the evaluation is time-consuming. The OABSS is a symptom-focused questionnaire developed by Homma and colleagues in Japan. Since it consists of just 4 questions, evaluation can be performed in a much shorter time than for the OAB-q. The OABSS was used in the present study to avoid placing too much of a burden on the subjects.

The prevalence of OAB pre-surgery in the present study was 4.1%, which was almost the same as that in women in the general population aged over 40 years reported in a previous study (8.1%) [26]. The present results showed, however, that the OAB rate increased significantly to 13.1% post-surgery, which is more than double that in the general population. Francesco et al. conducted urodynamic tests of 15 patients after total hysterectomy, and they reported the postoperative prevalence of OAB to be 27% [6]. It is difficult to directly compare the results of their study with those from the present study due to differences in the evaluation method; however, the results are consistent in terms of the rate of OAB increasing post-surgery. On the other hand, the present study showed that the proportion of those in whom OAB was recognized for the first time post-surgery was 10.8%. However, there are no previous reports on the proportion of newly developed UI post-surgery, so the results in this study represent a new finding.

The effects of aging are thought likely to be the major reason for OAB pre-surgery. The aging mechanisms thought to give rise to OAB include a decrease in the bladder relaxation response and a weakening of the pelvic floor due to disturbance of blood flow to the bladder [30]. However, some consideration should also be given to the possible compression of the bladder and autonomic nerves by the tumor associated with the primary disease, and the tumors were localized in all cases.

With regard to the development of OAB postoperatively, we considered that the parasympathetic pelvic nerve was dominant, because the sympathetic hypogastric nerve was damaged due to surgical stress from the invasive intervention, and it is therefore possible that α receptors led to the relaxation of the neck of the bladder, and β receptors induced contraction of the body of the bladder [31].

### Effects of UI/OAB on QOL

UI and OAB are both pathological conditions known to greatly impair QOL, particularly in women, who are known to experience adverse physical, emotional, and social effects. In a previous study that used the IIQ-7 to evaluate UI in 28 cervical cancer patients, the total score pre-surgery was 4.7 ± 0.8, and that at 6 months postoperatively was 10.9 ± 1.0, indicating that patient QOL was significantly worsened after surgery [32]. In the present study, IIQ-7 scores were compared by UI classification for patients with UI post-surgery. This is the first report of such a comparison, and the present results showed the scores for UI classifications to be in the order of mixed < urge < stress < overflow incontinence, with the IIQ-7 score for mixed incontinence being significantly lower than that for stress incontinence. The reason for this is that the frequency of UI in cases of stress incontinence can be reduced to a certain extent through one’s own behavior, whereas urgency cannot be controlled to a similar degree in cases of urge incontinence. A previous study that compared QOL by UI classification in the general female population reported that QOL for mixed incontinence was lower than that for stress incontinence, which supports our hypothesis.

Furthermore, all subscale scores were high for stress, urge, and mixed incontinence; however, for overflow incontinence, the subscale scores were high for travel/outing/social life, but zero (0) for physical activity and emotional impact. Overflow incontinence involves an increase in the volume of the bladder content, and this stored urine leaks out, resulting in “overflow”. The increased content can be compensated for by increasing the frequency of urination, so the patients consider the problem to be less severe, and the impact on their QOL is reduced. Based on these results, clinical improvement with pelvic floor muscle exercises, which are included in the first-line conservative management programs for UI [33], is desired from an early post-surgical stage in order to improve QOL. However, the relationships of many factors, such as the frequency and extent of physical activity, working conditions, and the ability to cope with UI, are yet to be established, and further study is needed.

### Risk factors for the onset of UI post-surgery

In the present study, the number of Cesarean sections and the days of urinary bladder catheterization post-surgery were identified as risk factors for UI post-surgery. Studies of the general female population showed that women with vaginal delivery have a higher frequency of UI than nullipara or those delivering by Cesarean section [34, 35]. It is thought that the increased rate of onset observed in the vaginal delivery group is due to neural damage to the pubococcygeus muscle during delivery [36] and injury to the pudendal nerve [37]. On the other hand, a study of UI in 505 pregnant women followed for 3 months after delivery showed that the incidence of UI was significantly lower in the Cesarean section group than in the vaginal delivery group. However, it was reported that there was no significant difference in the rate of UI between those in the vaginal delivery group and women having 3 or more Cesarean sections [35]. The fact that a higher number of Cesarean sections leads to a higher incidence of UI can be explained by the invasion of the abdominal wall during surgery. Repeated surgical invasion of the abdominal wall reduces the activity of the abdominal muscles, which then becomes unable to support the abdominal wall, resulting in lumbar lordosis. The condition in which the abdomen is extended due to the lumber lordosis acts to lower the pressure in the urethra, leading to UI [38]. Therefore, lumbar lordosis may need to be corrected for patients undergoing cesarean section.

With regard to the days of urinary bladder catheterization post-surgery, a longer period of catheterization can lead to urethral mucosal irritation or bladder irritation due to urinary tract infections. Such bladder irritation causes the bladder to suppress uncontrolled contractions, thereby resulting in urine leakage. Therefore, to reduce the number of bladder catheter days, it was considered clinically significant for patients to get out of bed early and to be able to use a regular toilet soon after surgery.

### Limitations and future issues

This study was conducted by postal questionnaire survey, with subjects responding to items covering frequency and volume of UI and the frequency of daytime and nighttime urination based on their situation pre-surgery, so the accuracy and reproducibility could be low. The proportion of women with UI increases with age and the number of vaginal deliveries [23]. Because the subjects analyzed were significantly older than the non-respondents, and the number of vaginal deliveries was also higher, these factors may have affected the results. The time between surgery and questionnaire response was quite long (mean 839.6 ± 48.6 days), and this could cause some bias with regard to the patients’ ability to recall pre-surgery symptoms or overvalue post-surgery symptoms. In addition, the necessary population size of the UI classification post-surgery of the subjects who had incontinence pre-surgery for this study calculated using G*Power version 3.1.9.4 for Windows was *n* = 48. However, the statistical analysis of the UI patients was based on a population of only 34, so that the potential for type 2 error cannot be excluded. Thus, it was not possible to conduct a meaningful subgroup analysis by cancer type. Furthermore, many of the patients who did not return their responses may have failed to do so due to feelings of shame about their current situation regarding UI. In order to accurately understand the situation regarding the onset and causes of UI pre-surgery, it is necessary to carry out future prospective studies to observe patients pre-surgery. Nevertheless, the present study identified patients who newly experienced UI post-surgery, and this is the first study to show such findings.

## Conclusions

The purpose of this study was to examine the rates and impact on QOL of urinary storage symptoms after gynecologic cancer surgery. UI and OAB rates were higher after surgery for gynecologic cancer than in the general female population, and this is the first study to identify patients newly experiencing UI post-surgery. The mixed incontinence rate was markedly higher post-surgery; QOL was low for such patients due to the combination of urge and stress incontinence. Multiple Cesarean sections and urinary bladder catheterization post-surgery were risk factors for post-surgical UI.

## Data Availability

The datasets during and/or analyzed during the current study are available from the corresponding author on reasonable request.

## Abbreviations

UI: Urinary incontinence
OAB: Overactive bladder
QOL: Quality of life
ICIQ-SF: International Consultation on Incontinence Questionnaire-Short Form
OABSS: Overactive Bladder Symptom Score
IIQ-7: Incontinence Impact Questionnaire-7
